# Effect of N Terminal Plasma Tau on Hippocampal Functional Connectivity in Alzheimer's Disease

**DOI:** 10.1002/alz70856_097272

**Published:** 2025-12-24

**Authors:** Jordan Williamson, Jack Lei Yang, Rhea Parekh, Yuan Yang

**Affiliations:** ^1^ University of Illinois Urbana‐Champaign, Urbana‐Champaign, IL, USA; ^2^ Carle Illinois College of Medicine, Urbana‐Champaign, IL, USA; ^3^ University of Illinois Urbana Champaign, Urbana, IL, USA

## Abstract

**Background:**

Functional alteration of the hippocampus is a critical consequence of Alzheimer's Disease (AD). The underlying pathological process has been attributed to the accumulation of amyloid‐beta plaques and protein tau as neurofibrillary tangles and is closely related to memory deficits. These changes in the hippocampus are known to emerge at the earliest stages of the disease, even before a diagnosis can be made. Functional connectivity measures, as well as brain levels of tau may be monitored by fMRI and CSF and PET biomarkers, respectively. However, these methods are hampered by their accessibility, relative invasiveness, or high costs. Blood‐based markers, such as plasma tau, may be an effective alternative. Therefore, we investigated the effect of plasma N Terminal (NT1)‐tau on hippocampal functional connectivity.

**Method:**

The resting state fMRI, T2 MRI, and plasma NT1‐tau levels of individuals with AD (*n* = 30, female=15) and cognitively normal individuals (*n* = 30, female=15) from the Alzheimer's Disease Neuroimaging Initiative (ADNI) were analyzed. Using the functional connectivity toolbox (CONN) a seed‐based connectivity map with the hippocampus selected as the region of interest was created for each participant. Correlation maps between plasma NT1‐tau and the functional connectivity of the hippocampus were compared.

**Result:**

Our findings revealed that higher plasma NT1‐tau values were associated with reduced hippocampal functional connectivity in AD compared to controls.

**Conclusion:**

The use of blood‐based markers has the potential of early AD detection and widespread clinical use, including in primary care. Future work involves increasing sample size, following the changes in longitudinal data, and including other plasma biomarkers such as amyloid beta.

